# Pathogenicity of Clinical OXA-48 Isolates and Impact of the OXA-48 IncL Plasmid on Virulence and Bacterial Fitness

**DOI:** 10.3389/fmicb.2019.02509

**Published:** 2019-11-01

**Authors:** Axel Hamprecht, Julian Sommer, Matthias Willmann, Christina Brender, Yvonne Stelzer, Felix F. Krause, Tsvetan Tsvetkov, Florian Wild, Sara Riedel-Christ, Julia Kutschenreuter, Can Imirzalioglu, Aitor Gonzaga, Ulrich Nübel, Stephan Göttig

**Affiliations:** ^1^Institute for Medical Microbiology, Immunology, and Hygiene, German Center for Infection Research (DZIF Partner Site Bonn–Cologne), University Hospital of Cologne, Cologne, Germany; ^2^Institute for Medical Microbiology and Infection Control, University Hospital, Goethe University Frankfurt am Main, Frankfurt, Germany; ^3^Institute of Medical Microbiology and Hygiene, German Center for Infection Research (DZIF Partner Site Tübingen), University of Tübingen, Tübingen, Germany; ^4^Institute of Medical Microbiology, German Center for Infection Research (DZIF Partner Site Gießen–Marburg–Langen), Justus-Liebig-Universität Gießen, Giessen, Germany; ^5^Leibniz Institute DSMZ – Deutsche Sammlung von Mikroorganismen und Zellkulturen, German Center for Infection Research (DZIF Partner Site Hannover–Braunschweig), Brunswick, Germany; ^6^Braunschweig Integrated Centre of Systems Biology, Technical University of Braunschweig, Brunswick, Germany

**Keywords:** OXA-48, carbapenemases, IncL, β-lactamases, virulence, fitness, plasmid

## Abstract

OXA-48 is the most common carbapenemase in Enterobacterales in Germany and one of the most frequent carbapenemases worldwide. Several reports have associated *bla*_OXA__–__48_ with a virulent host phenotype. To challenge this hypothesis, 35 OXA-48-producing clinical isolates of *Escherichia coli* (*n* = 15) and *Klebsiella pneumoniae* (*n* = 20) were studied *in vitro*, *in vivo* employing the *Galleria mellonella* infection model and by whole-genome sequencing. Clinical isolates belonged to 7 different sequence types (STs) in *E. coli* and 12 different STs in *K. pneumoniae*. In 26/35 isolates *bla*_OXA–__48_ was located on a 63 kb IncL plasmid. Horizontal gene transfer (HGT) to *E. coli* J53 was high in isolates with the 63 kb IncL plasmid (transconjugation frequency: ∼10^3^/donor) but low in isolates with non-IncL plasmids (<10^–6^/donor). Several clinical isolates were both highly cytotoxic against human cells and virulent *in vivo*. However, 63 kb IncL transconjugants generated from these highly virulent isolates were not more cytotoxic or virulent when compared to the recipient strain. Additionally, no genes associated with virulence were detected by *in silico* analysis of OXA-48 plasmids. The 63 kb plasmid was highly stable and did not impair growth or fitness in *E. coli* J53. In conclusion, OXA-48 clinical isolates in Germany are diverse but typically harbor the same 63 kb IncL plasmid which has been reported worldwide. We demonstrate that this 63 kb IncL plasmid has a low fitness burden, high plasmid stability and can be transferred by highly efficient HGT which is likely the cause of the rapid dissemination of OXA-48 rather than the expansion of a single clone or gain of virulence.

## Introduction

*Escherichia coli* and *Klebsiella pneumoniae* are the most common species causing Gram-negative sepsis (Goto et al., 2016). The increasing incidence of multidrug-resistant (MDR) *E. coli* and *K. pneumoniae* strains has complicated the treatment of Gram-negative sepsis and other invasive infections. Among MDR bacteria, carbapenemase-producing Enterobacterales (CPE) are a major threat to our health system, because of resistance to most or all β-lactam antibiotics and a lack of therapeutic options. As a consequence, the mortality in patients infected with CPE is 26–44% higher than in patients infected with carbapenem-susceptible isolates (Falagas et al., 2014).

Among the different carbapenemases, OXA-48 has been particularly successful and is the most common carbapenemase in Germany. It has rapidly disseminated from Europe and the Middle East to every continent (Canton et al., 2012). OXA-48 is most frequently detected in *K. pneumoniae* and *E. coli*, but can also occur in other Enterobacterales (Khajuria et al., 2013; Manageiro et al., 2014). The β-lactamase gene (*bla*) encoding OXA-48 (*bla*_OXA–__48_) is usually located on plasmids (pOXA-48) and bracketed by two identical insertion sequences, IS*1999*, forming the composite transposon Tn*1999* (Giani et al., 2012; Poirel et al., 2012). Different variants of OXA-48, commonly referred to as OXA-48-like (e.g., OXA-181, OXA-232), have been described which differ from OXA-48 by several amino acids, but have not spread as successfully as OXA-48 (Lascols et al., 2013). However, the reasons for the successful dissemination of OXA-48 are currently not fully understood.

Interestingly, virulence has been linked to the presence of *bla*_OXA__–__48_ in clinical *E. coli* and *K. pneumoniae* isolates. Several studies reported on clinical isolates with an unusual high lethality in murine infection models as well as the presence of genes associated with virulence or host colonization, but the specific role of OXA-48 had not been addressed (Beyrouthy et al., 2013, 2014; de Toro et al., 2017). Since the dissemination is not driven by a single sequence type (ST), it is possible that OXA-48 itself or *bla*_OXA–__48_ harboring plasmids contribute to fitness and virulence of clinical isolates which would provide a survival benefit. We therefore investigated if acquisition of *bla*_OXA__–__48_ carrying plasmids affects fitness or virulence of *E. coli* and *K. pneumoniae*. For this purpose, OXA-48 producing clinical isolates of *E. coli* and *K. pneumoniae* were analyzed by whole genome sequencing (WGS) including single molecule real time sequencing (SMRT). Additionally, factors involved in dissemination and virulence were investigated *in silico*, *in vitro*, and *in vivo*.

## Materials and Methods

### Clinical Isolates

A total of 35 clinical, non-copy *bla*_OXA__–__48_ carrying *E. coli* and *K. pneumoniae* strains were included, which were isolated in 2010–2017 in two large German tertiary care hospitals (University Hospitals of Cologne and Frankfurt). All isolates were phenotypically characterized and tested for *bla*_OXA–__48_ by PCR and Sanger sequencing as described previously (Göttig et al., 2015; Hamprecht et al., 2016; Koroska et al., 2017). Minimal inhibitory concentrations (MIC) were determined by MIC test strips (Liofilchem, Roseto degli Abruzzi, Italy) and interpreted according to CLSI breakpoints. Multilocus sequence typing (MLST) was performed either by PCR employing the Pasteur scheme^1^ or by WGS using the CGE Sequence Typer Tool.

### Horizontal Gene Transfer and Analysis of *bla*_OXA__–__48_ Plasmids

Horizontal gene transfer (HGT) of *bla*_OXA__–__48_ was evaluated by liquid mating assays using clinical *Enterobacteriaceae* isolates as donors and sodium azide-resistant strains *E. coli* J53 (EC-J53) and *K. pneumoniae* PRZ (KP-PRZ), *Serratia marcescens* PRaT, *Enterobacter cloacae* DdL, and *Citrobacter freundii* ULTN as recipients as described (Hamprecht et al., 2013; Gruber et al., 2015). Transconjugants (Tc) were selected employing chromogenic agar plates containing 20 μg/mL amoxicillin-clavulanate (ACA) and 100 μg/mL sodium azide. Transconjugation frequency was determined by dividing the numbers of Tc by the numbers of donors. Estimation of the *bla*_OXA__–__48_ carrying plasmid size was done by S1 nuclease digestion and pulsed-field gel electrophoresis (PFGE). PCR and Sanger sequencing was employed to determine the incompatibility group of OXA-48 plasmids (Carattoli et al., 2015) and the Tn*1999* transposon variants (Supplementary Table 1).

### Next Generation Sequencing and Data Analysis

Whole genome sequencing was carried out using the Illumina MiSeq platform applying a v3 reagent kit (Illumina, San Diego, CA, United States) generating 150 or 250 bp paired-end reads with coverage of ≥50. *De novo* assembly and scaffolding after quality trimming of the reads was conducted using SPAdes v3.12.0 with standard parameters (Bankevich et al., 2012). To generate complete genome sequences, SMRT long-read sequencing (Pacific Biosciences, Menlo Park, CA, United States) in combination with Illumina short-read sequencing was applied in selected isolates as described (Steglich et al., 2018).

Genomes were annotated using Prokka 1.8 software (Seemann, 2014), and annotation was corrected manually. Genome sequences were visualized using the BRIG-Software and SnapGene^®^ Viewer (Alikhan et al., 2011). Sequence comparison of plasmids was performed and visualized using an in-house Perl script utilizing BLAST. Short reads of isolates harboring the 63 kb IncL plasmid were mapped to the plasmid sequence from EC7215 (NZ_LR025098.1) used as a reference employing Bowtie2 and visualized using Geneious 11.1.5^2^ (Langmead and Salzberg, 2012). Fully closed genome sequences were submitted to NCBI GenBank and can be found under accession number LR025099–LR025100 (KP12536) and LR025096–LR025098 (EC7215). Screening for virulence genes was performed using *K. pneumoniae* BIGS database, VFDB^3^ and a database which was created based on selected publications (Supplementary Figures 1, 2). Antimicrobial resistance genes were identified using the tool ABRicate^4^ applying CGE ResFinder database (Zankari et al., 2012).

### Determination of Cytotoxicity

A549 human lung epithelial cells (ATCC^®^ CCL-185) were grown in RPMI medium (Biochrom GmbH, Berlin, Germany) with 10% fetal calf serum (FCS) (Sigma, Taufkirchen, Germany) at 37°C until almost confluent and infected with bacteria at a multiplicity of infection (MOI) of 100. After 24 h, the supernatant was filtered (0.45 μm) and the lactate dehydrogenase (LDH) activity was determined by spectrophotometry (Cobas 8000, module 701; Roche, Grenzach-Wyhlen, Germany).

### Evaluation of *in vivo* Virulence Using the *Galleria mellonella* Infection Model

Larvae of the greater wax moth (*G. mellonella*) were obtained from UK Waxworms (Sheffield, United Kingdom). Infection of larvae, read-out, and controls were performed as previously described (Göttig et al., 2016). Median lethal doses (LD_50_) were calculated by non-linear regression analysis using GraphPad Prism 5.0 (La Jolla, CA, United States) (Gruber et al., 2015).

### Evaluation of Bacterial Fitness and Plasmid Stability

For analysis of non-competitive growth kinetics, brain heart infusion (BHI) broth (Becton Dickinson, Heidelberg, Germany) were inoculated with an overnight culture and incubated at 37°C and shaking until an optical density of 0.5 at 600 nm (OD_600_) was reached. Bacteria were adjusted to an OD_600_ of 1 in phosphate-buffered saline (PBS), serially diluted and inoculated into an aerobe BD Bactec bottle (BD, Heidelberg, Germany), resulting in a final inoculum of 10^3^ colony-forming units (cfu) per bottle. Bacterial growth was recorded for 24 h at 37°C in a Bactec Fx instrument (BD) by measuring the fluorescence intensity every 15 min. The doubling time was calculated using MS Excel and the Solver tool.

To assess the impact of *bla*_OXA__–__48_ carriage on bacterial fitness, pairwise competition assays with Tc carrying the natural plasmid pOXA-48 from EC7215 were employed. EC-J53 Tc7215 and its parental strain EC-J53 were analyzed as described before (Lenski et al., 1994; Göttig et al., 2016). For determination of cfu, serial dilutions of each culture were plated on Mueller Hinton agar (MHA) and MHA containing 12 mg/L ACA (MHA-ACA). Selection rate constant (s) was calculated as described by Lenski et al. (1994) with negative *s*-values indicating a loss of fitness. To estimate plasmid stability, a pure culture of EC-J53 Tc_7215_ and KP-PRZ Tc7215 was cultured under the same conditions as for the pairwise competitions experiments in antibiotic-free medium. The ratio of colonies growing on MHA-ACA compared to antibiotic-free MHA was determined after three transfers at 90 h in triplicate.

### Statistical Analysis

Categorical variables were compared using the Chi-square or Fisher’s exact test where appropriate. Continuous variables were assessed by Mann–Whitney *U* test. A *P*-value of <0.05 was considered significant.

### Ethics Statement

All bacterial strains were isolated as part of routine microbiological diagnostic and stored in an anonymized database. According to the ethic committee of the Hospital of Johann Wolfgang Goethe-University, Frankfurt am Main, no informed consent or ethical approval of the study is necessary.

## Results

### Whole-Genome Sequencing and Molecular Characterization of OXA-48 Producing Clinical Isolates

Thirty-five non-copy clinical isolates producing OXA-48 including 15 *E. coli* and 20 *K. pneumoniae* were characterized (Tables 1, 2). For *E. coli*, ST38 (*n* = 5) and ST354 (*n* = 4) were the most frequent STs. *E. coli* isolates harbored *bla*_OXA–__48_ on a mobilizable plasmid in 10/15 cases. In 6/15 isolates *bla*_OXA–__48_ was embedded in a Tn*1999* structure which was located on a 63 kb IncL plasmid, whereas one isolate harbored an IncL plasmid with a size of 75 kb. Three isolates carried IncF plasmids of different sizes (80–89 kb). In five isolates, *bla*_OXA–__48_ could be transferred neither by conjugation nor by electroporation, indicating a chromosomal location, which was confirmed by WGS and PCRs amplifying Tn*6237* embedded *bla*_OXA–__48_ and the adjacent chromosomal genetic environment (Turton et al., 2016).

**TABLE 1:** Characteristics of OXA-48 harboring *E. coli* isolates.

**Isolate**				**Genetic support of OXA-48**			**MIC (mg/L)**					
**Isolate^a^**	**ST (Warwick)**	**ST (Pasteur)**	**Specimen**	**Transposon^b^**	**Plasmid size**	**IncL**	**CTX**	**CAZ**	**ETP**	**IMP**	**MEM**	**CIP**
**EC1027**	297	666	Rectal swab	Tn*1999.2*	63 kb	+	256^H^	16	>32	>32	16	>32
**EC1639**	393	494	Blood	invTn*1999.2*	63 kb	+	1	0.25	4	4	0.5	>32
**EC2058**	38	8	Rectal swab	Tn*6237*	Chromosomal	–	64	16	2	0.5	0.25	0.25
**EC2196**	38	8	Rectal swab	Tn*6237*	Chromosomal	–	64	2	16	8	2	0.25
**EC2269**	38	8	Rectal swab	ΔTn*6237*	Chromosomal	–	64	1	2	2	2	0.25
**EC2667**	354	39	Rectal swab	Tn*1999*	63 kb	+	>256	>256	4	4	2	>32
EC2700	354	39	Rectal swab	Tn*1999*	63 kb	+	256^H^	256^H^	16	16	2	>32
**EC3124**	38	8	Rectal swab	Tn*6237*	Chromosomal	–	256^H^	2	4	4	1	0.25
EC3239	10	262	Bile fluid	invTn*1999.2*	75 kb	+	4	0.5	4	4	2	>32
**EC3338**	354	39	Rectal Swab	ΔTn*6237*	87 kb	–	256^H^	4	2	1	0.25	>32
EC3428	38	8	Rectal swab	Tn*6237*	Chromosomal	–	32	1	2	4	1	0.25
EC3471	1598	398	Rectal swab	Tn*1999.2*	63 kb	+	32	64	1	2	0.25	0.5
**EC7215**	448	58	Rectal swab	Tn*1999.2*	63 kb	+	0.5	0.25	1	1	0.5	>32
**EC9629**	354	39	Blood	ΔTn*6237*	80 kb	–	64	2	1	1	0.5	>32
EC14604	1598	398	Rectal swab	ΔTn*6237*	89 kb	–	>256	16	0.25	0.5	0.12	0.25

*H, heteroresistance; CTX, cefotaxime; CAZ, ceftazidime; ETP, ertapenem; IMP, imipenem; MEM, meropenem; CIP, ciprofloxacin; ^*a*^Isolates which were further assessed in virulence assays are highlighted in bold. ^*b*^Tn*6237* refers to KT444705 (Genbank); ΔTn*6237* of EC2269, EC3338, EC9629, and EC14604 refer to a 19.9 kb fragment of KT444704, KT444705 (Genbank), 3.3 kb fragment of KT444704, and 21.5 kb fragment of KT444705, respectively.*

**TABLE 2:** Characteristics of OXA-48 harboring *K. pneumoniae* isolates.

**Isolate**			**Genetic support of OXA-48**			**MIC (mg/L)**					
**Isolate^a^**	**ST (Pasteur)**	**Specimen**	**Transposon**	**Plasmid size**	**IncL**	**CTX**	**CAZ**	**ETP**	**IMP**	**MEM**	**CIP**
KP659	15	Stool	Tn*1999.2*	63 kb	+	>256	32	>32	0.5	2	>32
**KP920**	16	Wound swab	Tn*1999.2*	63 kb	+	>256	>256	>32	>32	>32	>32
**KP980**	16	Rectal swab	Tn*1999.2*	63 kb	+	>256^H^	16	>32	2	2	>32
KP1151	15	Rectal swab	Tn*1999.2*	63 kb	+	>256	>256	>32	>32	>32	>32
**KP1664**	16	Throat swab	Tn*1999.2*	63 kb	+	256^H^	64	32	32	32	>32
KP1673	101	Urine	Tn*1999.2*	63 kb	+	>256	>256	>32	32	>32	>32
KP1696	377	Skin swab	invTn*1999.2*	63 kb	+	>256	>256	3	1	1	>32
KP1766	1658	Skin swab	invTn*1999.2*	63 kb	+	>256	>256	>32	16	32	>32
**KP1982**	377	Wound swab	Tn*1999.2*	63 kb	+	>256	>256	32	8	16	>32
**KP2255**	437	Rectal swab	invTn*1999.2*	63 kb	+	>256	128	32	32	32	>32
KP2451	392	Rectal swab	Tn*1999.2*	63 kb	+	>256	>256	2	16	4	>32
KP2540	392	Rectal swab	invTn*1999.2*	63 kb	+	64	128	2	4	2	> 32
**KP2575**	3384	Rectal swab	Tn*1999*	63 kb	+	4	2	>32	8	8	0.06
**KP2670**	101	Tracheal secretion	Tn*1999*	63 kb	+	0.5	0.25	2	2	1	0.03
**KP2746**	101	Liver biopsy	invTn*1999.2*	63 kb	+	2	0.5	16	8	4	0.03
KP3379	307	Urine	Tn*1999.2*	63 kb	+	2	1	4	2	4	16
KP3907	307	Stool	Tn*1999.2*	63 kb	+	>256	32	>32	2	1	>32
**KP12536**	3385	Tracheal secretion	Tn*1999.2*	63 kb	+	0.5	0.25	16	2	4	0.03
**KP12883**	323	Urine	invTn*1999.2*	63 kb	+	0.5	0.25	1	8	1	>32
KP13815	11	Wound swab	Tn*1999.2*	63 kb	+	>256	>256	2	4	2	>32

*H, heteroresistance; CTX, cefotaxime; CAZ, ceftazidime; ETP, ertapenem; IMP, imipenem; MEM, meropenem; CIP, ciprofloxacin; ^*a*^Isolates which were further assessed in virulence assays are highlighted in bold.*

In *K. pneumoniae* isolates, STs varied considerably (12 different STs). Strikingly, in all isolates *bla*_OXA–__48_ was located on an IncL plasmid with a size of ∼63 kb and embedded in a Tn*1999* family transposon (Table 2). Tn*1999.2* was the most common transposon variant (*n* = 12), followed by its inverted version invTn*1999.2* (*n* = 6) and two isolates harboring Tn*1999*.

In 30 of 35 isolates, *bla*_OXA–__48_ was identified on plasmids of which 26 were 63 kb IncL plasmids. Of these, five plasmids were completely sequenced by both Illumina^®^ short-read and SMRT long-read technology revealing a sequence identity of each ≥99.9%. To analyze the 63 kb IncL plasmids of the 21 isolates, which were solely sequenced by short-read technology, the sequencing reads of each isolate were mapped to the complete sequence of the 63 kb plasmid IncL from EC7215 (Brehony et al., 2019). Thereby, a median coverage of >50 of each isolate to the reference sequence from EC7215 was noticed, suggesting the presence of highly similar plasmids in all these isolates (Supplementary Figure 1). The IncL plasmid from EC3239 had a length of 75,552 bp, a sequence identity of >99% compared to the 63 kb IncL plasmids and carried an additional 8.3 kb Tn*3* transposon and a 4.4 kb fragment of a Col440I plasmid (highest sequence homology to CP039328.1 and AF527822.1, respectively).

The three isolates EC14604, EC3338, and EC9629 harbored *bla*_OXA–__48_ on IncF plasmids of different sizes (80–89 kb). The sequences of the plasmids from EC3338 and EC9629 were highly similar and both plasmids shared the same IncF type F-:A1:B32. In contrast, the *bla*_OXA–__48_ plasmid from EC14604 had a different IncF type (F2:A-:B-) and displayed a lower sequence similarity compared to the two other EC isolates (Supplementary Figure 2). The transposon structures containing *bla*_OXA–__48_ on these three IncF plasmids were more diverse compared to the IncL plasmids. The plasmid from EC14604 harbored a 21,571 bp fragment of Tn*6237* in which one of the two copies of the flanking IS*1R* mobile elements was truncated by a Tn*3*-type and a IS*6*-like type insertion element. In EC3338, the *bla*_OXA–__48_ plasmid harbored a 12,192 bp fragment of Tn*6237*, flanked on one side by a complete 767 bp IS*1R*-element and a 472 bp fragment of an IS*1R*-element on the other side. The IncF plasmid from isolate EC9629 harbored only a short fragment of Tn*6237* consisting of two copies of IS*1R* sequences up and downstream of *bla*_OXA–__48_ and a truncated *lysR* gene.

Antimicrobial susceptibility testing revealed highly variable MICs for carbapenems with meropenem MICs ranging from 0.125 to 16 mg/L in *E. coli* and from 1 to >32 mg/L in *K. pneumoniae*. Ceftazidime remained susceptible in 8/15 *E. coli* and 4/20 *K. pneumoniae* isolates. Ciprofloxacin resistance was common in both *E. coli* (8/15) and *K. pneumoniae* (16/20). WGS revealed that 8 of 15 *E. coli* isolates carried genes encoding ESBLs, with CTX-M-24 being the most frequent (7/9), and two isolates expressing CMY-42 AmpC β-lactamases, which is in line with the resistance phenotype (Supplementary Table 2). Interestingly, CTX-M-15 was present in 13/14 ESBL-producing *K. pneumoniae* isolates but detected in only one strain of *E. coli*, whereas CTX-M-24 was found only in *E. coli*.

Taken together, clinical OXA-48 isolates from Germany are diverse, but mostly carry *bla*_OXA__–__48_ on a 63 kb sized IncL plasmid. In *K. pneumoniae bla*_OXA__–__48_ was exclusively linked to a Tn*1999* type transposon and the 63 kb sized IncL plasmid. In *E. coli*, genetic support of *bla*_OXA–__48_ was more diverse, but the same 63 kb plasmid was detected in half of the isolates. From the 35 clinical isolates, ten representative *K. pneumoniae* and *E. coli* each were selected based on different criteria (ST type, transposon type, isolation site, and MIC) to study HGT, virulence, and fitness.

### Horizontal Gene Transfer of *bla*_OXA–__48_

**FIGURE 1:**
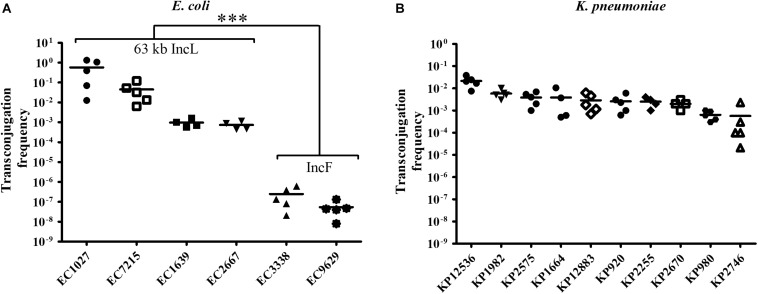
Transconjugation frequency during horizontal transfer of plasmids harboring *bla*_OXA–48_ from *E. coli* and *K. pneumoniae* clinical isolates to EC-J53. Each dot represents the frequency of a single transconjugation experiment, whereas the bars indicate the arithmetic mean of all experiments. **(A)** Transconjugation frequency from *E. coli* (EC) donors. The first four EC isolates harbor a 63 kb IncL *bla*_OXA–48_ plasmid with a Tn*1999* type plasmid (“63 kb IncL”), whereas EC3338 and EC9629 contain an IncF plasmid. ^∗∗∗^*P* < 0.0001 (Mann–Whitney *U* test). **(B)** Transconjugation frequency from *K. pneumoniae* (KP) donors.

To investigate HGT efficiency of pOXA-48, liquid mating assays were performed using *E. coli* isolates as donors and sodium azide-resistant EC-J53 as a recipient. Except for the five *E. coli* isolates with chromosomal localization of *bla*_OXA–__48_, pOXA-48 could be transferred indicating successful intraspecies transconjugation. The conjugation frequency was high for all donors carrying the 63 kb IncL plasmid (median frequency: 1.7 × 10^–1^ ± 3.8 × 10^–1^). In contrast, a significantly lower frequency of 1.5 × 10^–7^ ± 1.9 × 10^–7^ was determined for the isolates with IncF plasmids (*P* < 0.0001, Figure 1A). In *K. pneumoniae*, pOXA-48 could be transferred to EC-J53 in all cases with a high conjugation frequency of 4.7 × 10^–3^ ± 7.1 × 10^–3^, indicating efficient intergenus transconjugation (Figure 1B). The 63 kb IncL plasmid could furthermore be conjugated from *E. coli* and *K. pneumoniae* into other sodium azide-resistant Enterobacterales species including *K. pneumoniae* PRZ, *S. marcescens* PRaT, *E. cloacae* DdL and *C. freundii* ULTN with mean transconjugation frequencies of 6.8 × 10^–3^–1.3 × 10^–6^ (Supplementary Figure 3).

These results suggest that the 63 kb IncL *bla*_OXA–__48_ plasmid occurs in different *K. pneumoniae* and *E. coli* lineages, has a broad host range in Enterobacterales and can be mobilized by efficient intraspecies and intergenus HGT with a high frequency of up to 5.7 × 10^–1^.

### Virulence of OXA-48 Producing Clinical Isolates

**FIGURE 2:**
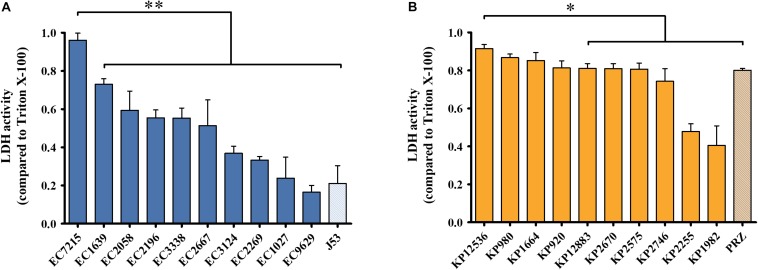
Cytotoxicity of OXA-48 isolates. **(A)** *E. coli* and **(B)** *K. pneumoniae*. LDH activity was measured from supernatants of infected A549 lung epithelial cells. Mean values from four independent experiments were compared to that of Triton X-100 which served as the positive control. Error bars represent standard deviations. ^∗∗^*P* < 0.0001; ^∗^*P* < 0.05 (Mann–Whitney *U* test).

To evaluate virulence traits of clinical OXA-48 producing isolates, cytotoxicity toward human host cells and virulence in an *in vivo* infection model was investigated. First, human lung epithelial cells were infected with the 20 selected *E. coli* and *K. pneumoniae* isolates and the release of the intracellular enzyme LDH was measured as a surrogate marker for cytotoxicity (Figure 2). After infection with *E. coli* isolates, the mean LDH activity compared to the positive control was 37.8 ± 24.1% (Figure 2A). EC7215 was significantly more cytotoxic than all other strains (*P* < 0.0001). LDH activity was high after infection with *K. pneumoniae* (mean: 76.0 ± 18.6%), indicating high species-specific membrane disintegration of *K. pneumoniae* compared to *E. coli* (*P* < 0.0001). KP12536 was the most cytotoxic *K. pneumoniae* isolate, whereas KP2255 and KP1982 were significantly less cytotoxic compared to all other isolates (*P* < 0.05).

**FIGURE 3:**
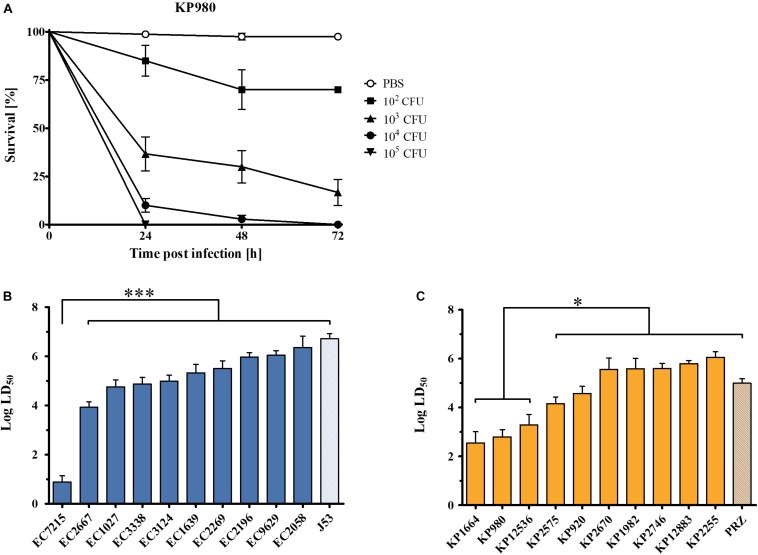
Virulence of OXA-48 producing *Enterobacteriaceae* clinical isolates in the *Galleria mellonella in vivo* infection model. **(A)** Dose-dependent mortality of *G. mellonella* infected with KP980. Different cfu were injected into the larvae and survival was monitored for 72 h. Error bars show standard error of the mean. **(B)** Log median lethal dose (LD50) of clinical *E. coli* isolates. **(C)** LD50 of *K. pneumoniae* isolates. Mean values from three to four independent experiments with 12 larvae in each group are shown. Error bars represent the 95% confidence interval. ^∗∗∗^*P* < 0.0001; ^∗^*P* < 0.05 (Mann–Whitney *U* test).

To assess pathogenicity *in vivo*, the *G. mellonella* infection model was employed. To compare virulence across the isolates, time-kill curves were compared and median lethal dose (LD_50_) values were calculated. All *E. coli* and *K. pneumoniae* isolates caused a time- and dose-dependent killing of larvae (Figure 3A). The mean LD_50_ was 4.9 for *E. coli* (range: 0.9–6.4) and 4.6 for *K. pneumoniae* (range: 2.5–6.1). Among *E. coli* isolates, EC7215 was highly virulent [LD_50_ 0.9 (95% CI 0.63–1.14)] compared to all other clinical strains and the reference strain J53, which was the least virulent [LD_50_ 6.7 (95% CI 6.50–6.91)]. In *K. pneumoniae*, the isolates KP980, KP1664, and KP12536 were significantly more virulent (LD_50_ 2.5–3.3) than the other clinical isolates and KP-PRZ (*P* < 0.05). In summary, EC7215 and KP12536 were highly virulent in both assays (Figures 2, 3).

### *In silico* Analysis of Virulence

**FIGURE 4:**
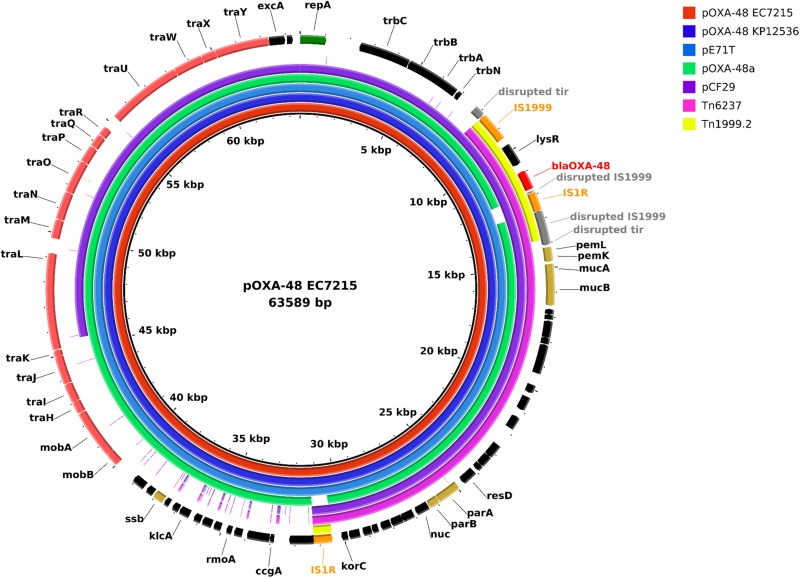
Plasmid map of pOXA-48 and comparison of plasmids harboring *bla*_OXA–48_ and the transposons Tn*1999.2* and Tn6237. Each ring represents the nucleotide sequence of a plasmid or the transposon Tn6237 as indicated by the color code. Sequences of pE71T (accession no. KC335143.1), pOXA-48a (JN626286.1), pCF29 (LN864820.1), and Tn6237 (HG977710.1) were retrieved from the NCBI nucleotide database. Gene annotation of the reference plasmid pOXA-48 EC7215 is shown in the outer ring. Tn*1999.2* indicates the transposon region on all plasmids in this graph. Colored arrows indicate *bla*_OXA__–__48_ (red), insertion elements (orange), genes for plasmid stabilization (gold), and replication (green), disrupted genes (gray) and genes of unknown function (*bla*ck).

To investigate virulence mediated by the *bla*_OXA–__48_ carrying plasmids *in silico*, the two highly virulent isolates EC7215 and KP12536 were sequenced by SMRT to obtain the complete sequence of the plasmids. The chromosome of EC7215 harbored a genome of 4,830,938 bp and a GC content of 50.8%, compared to 5,323,688 bp and 57.5% in KP12536. Both isolates contained an identical *bla*_OXA–__48_ encoding plasmid of 63,589 bp (Figure 4), which showed a 99.9% similarity to strain KPN-El-Nr.7 (Carattoli et al., 2015). The plasmid contained 41 genes and 28 open reading frames (ORF) encoding hypothetical proteins of unknown function. Among the predicted genes located on the plasmid, no virulence gene could be detected by any of the virulence gene databases (see section “Materials and Methods”).

Whole genome analysis demonstrated that *E. coli* isolates harbored 70–122 genes associated with virulence, while *K. pneumoniae* isolates possessed 42–59 virulence genes and were less diverse regarding the presence of virulence genes (Supplementary Figures 1, 2). Analysis of all thirteen isolates sequenced by SMRT showed that the vast majority of virulence genes was located on the chromosome, whereas only five different virulence genes were identified on plasmids which, however, did not harbor *bla*_OXA–__48_. *E. coli* isolates harbored genes encoding virulence factors of different cellular function, e.g., adhesion, invasion, effector protein secretion, and the pathogenicity island PAIII_CFT__073_. The virulence factors *wabG* and *uge*, which are important for host colonization and virulence and *kfu* which is involved in ion homeostasis, were present in all *K. pneumoniae* clinical isolates. Twenty-one virulence genes were detected in all isolates, e.g., the adhesin *focA* or the toxin *astA* which is commonly found in enterohemorrhagic *E. coli* (EHEC) and enteroaggregative *E. coli* (EAggEC). The mean number of virulence genes was 101 in *E. coli* and 47 in *K. pneumoniae* isolates. The isolates with the highest virulence in the *Galleria* model, *E. coli* EC7215 (86 virulence genes) and *K. pneumoniae* KP1664 (42 virulence genes) did not show higher numbers or distinct patterns of virulence genes compared to other isolates of the same species. In contrast, more virulence genes were found in EC1639 (122 virulence genes), which was isolated from a blood stream infection, but which neither showed a higher cytotoxicity nor a lower LD_50_ compared to the other isolates.

### Virulence and Fitness of *bla*_OXA–__48_ Harboring Transconjugants

The plasmid pOXA-48 EC7215 contained 28 ORFs of unknown function which potentially could encode for proteins mediating virulence or fitness. Therefore, transconjugants carrying *bla*_OXA–__48_ (Tc) were used to investigate the impact of pOXA-48 on pathogenicity and fitness and compared to their isogenic *E. coli* and *K. pneumoniae* parental strains.

**FIGURE 5:**
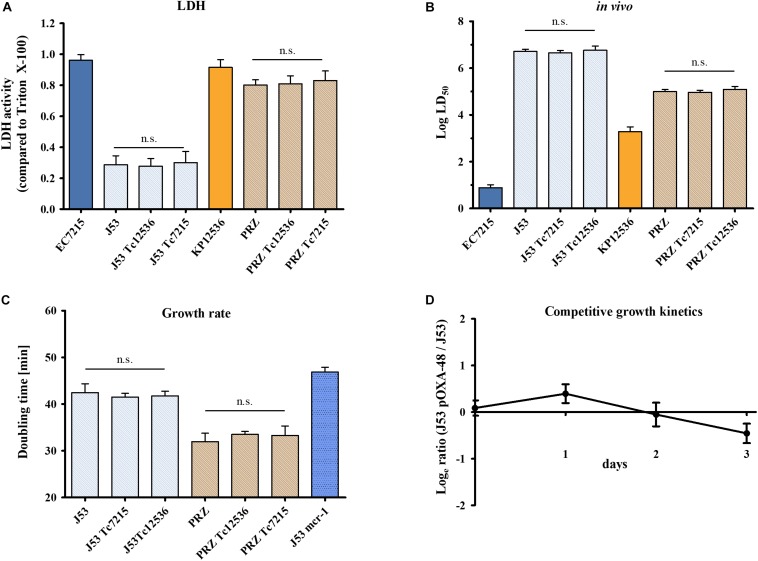
Impact of pOXA-48 carriage on virulence and fitness. Cytotoxicity, virulence, and growth kinetics of clinical isolates EC7215 and KP12536 harboring pOXA-48, acceptor strains J53 and PRZ, and pOXA-48 transconjugants (Tc) were analyzed. **(A)** Cytotoxicity was determined by the LDH assay and **(B)** virulence by *G. mellonella* infection experiments as described above. **(C)** Doubling times were calculated from growth kinetics. An EC-J53 control strain (“J53 mcr-1”) harboring the plasmid pKP2442-mcr-1, whose carriage causes a fitness loss, was used as a control (Tietgen et al., 2018). Bars represent the mean (*n* = 4). Error bars denote standard error **(A,C)** or the 95% CI **(B)**. n.s., no statistically significant differences as determined by the Mann–Whitney *U* test. **(D)** Dynamics of replicate competition experiments for EC-J53 and transconjugants containing the natural plasmid pOXA-48. The ratio of resistant versus susceptible cfu is shown (mean values from four independent experiments). Error bars denote the standard deviation of mean cfu counts.

The infection of A549 cells with EC-J53 and J53 transconjugants harboring pOXA-48 from EC7215 (J53 Tc7215) and from KP12536 (J53 Tc12536) caused a LDH activity of 0.29 ± 0.06, 0.28 ± 0.05, and 0.30 ± 0.07, respectively, thereby showing no effect of pOXA-48 carriage on cytotoxicity (Figure 5A). Likewise, carriage of pOXA-48 in *K. pneumoniae* Tc and parental strains did not alter cytotoxicity toward A549 cells. In comparison, the clinical strains EC7215 (LDH 0.96 ± 0.04) and KP12536 (LDH 0.92 ± 0.05) were significantly more cytotoxic than all transconjugants (*P* < 0.01).

To further explore virulence mediated by OXA-48, *G. mellonella* larvae were infected with transconjugants and their isogenic parental strains (Figure 5B). LD_50_ values were similar for EC-J53 Tc (log LD_50_ = 6.65 ± 0.10 and 6.76 ± 0.18) and the parental strain (log LD_50_ = 6.71 ± 0.10), *P* = 0.74, and *P* = 0.98, respectively. Likewise, carriage of pOXA-48 in KP-PRZ did not have a significant impact on LD_50_ values, indicating no substantial effect of *bla*_OXA–__48_ expression on virulence in *G. mellonella* (*P* = 0.85 and *P* = 0.73). In summary, the acquisition of pOXA-48 did not result in higher cytotoxicity or *in vivo* virulence.

### Impact of pOXA-48 on Bacterial Fitness

To analyze the impact of the acquisition of pOXA-48 on fitness, growth kinetics were compared between EC-J53 Tc7215 and Tc12536 and their parental strains (Figure 5C). Doubling times were 42.4 ± 1.9 min for J53, 41.5 ± 0.8 min for J53 Tc7215, and 41.8 ± 1.0 min for J53 Tc12536 (*P* > 0.05). In contrast, transconjugants harboring the plasmid pKP2442 containing the colistin resistance gene *mcr-1* had a doubling time of 46.9 min (*P* = 0.0095), indicating a loss of fitness (Tietgen et al., 2018). In KP-PRZ, transconjugants grew as fast as the parental strain (doubling times: 32.0 ± 1.8 min for PRZ, 33.5 ± 0.6 min for PRZ Tc12536, and 33.3 ± 2.0 min for PRZ Tc7215, *P* > 0.05). In conclusion, acquisition of pOXA-48 did not have an impact on the doubling time when compared to the parental strains.

In addition, pairwise competitive growth experiments using minimal media were performed to further assess the impact of pOXA-48 on bacterial fitness. For this purpose, *E. coli* transconjugant J53 Tc7215 competed with its isogenic parental strain EC-J53 (Figure 5D) and only a minor impact on fitness was recorded in the absence of antibiotics (selection rate constant s = −0.18 ± 0.02).

To assess plasmid stability, the plasmid loss of transconjugants EC-J53 Tc7215 and KP-PRZ Tc7215 was assessed by serial subcultures in antibiotic-free medium. In *E. coli*, the ratio of cells with and without plasmid was 1.026 ± 0.035 and 0.999 ± 0.156 in KP-PRZ, indicating a stable insertion of pOXA-48 with no plasmid loss.

## Discussion

The successful dissemination of OXA-48 producing isolates is currently not completely understood. In contrast to other successful β-lactamase producing clonal lineages (e.g., *K. pneumoniae* KPC-2 ST258 or *E. coli* CTX-M-15 ST131), OXA-48 isolates belong to different STs but frequently harbor similar plasmids (Beyrouthy et al., 2013; Potron et al., 2013). In this study, 35 OXA-48 producing *E. coli* and *K. pneumoniae* clinical isolates were molecularly characterized and factors involved in dissemination and virulence were investigated *in silico*, *in vitro*, and *in vivo*.

Antibiotic susceptibility, resistance determinants and STs were highly diverse in the isolates investigated and did not correlate with virulence properties. In contrast, all *K. pneumoniae* and the majority of *E. coli* isolates harbored the same *bla*_OXA–__48_ encoding 63 kb IncL plasmid, which was highly transmissible in both intraspecies and intergenus transconjugation assays. This plasmid was highly similar to the first isolate of OXA-48 in Turkey in 2001 (Poirel et al., 2004) and the isolates KPN-El-Nr.7 from Switzerland (Carattoli et al., 2015). In contrast to the first description with Tn*1999* in 2001, most of our clinical isolates harbored a Tn*1999.2* transposon. Isolates with Tn*1999.2* have been reported to hydrolyze imipenem at higher rates than isolates carrying Tn*1999* due to increased expression of *bla*_OXA–__48_ (Carrer et al., 2008). Genetic support of *bla*_OXA–__48_ was more diverse in *E. coli* with *bla*_OXA–__48_ being located on the chromosome or on different plasmids including non-IncL variants. These non-IncL plasmids could be mobilized with much lower frequencies of only 10^–7^ compared to 10^–3^ for IncL plasmids, indicating that dissemination of *bla*_OXA–__48_ is facilitated by the 63 kb sized IncL plasmid. The OXA-48 transposon structures on these non-IncL plasmids consisted of fragments of the chromosomally located Tn*6237*. However, IS*1R* sequences were truncated by additional mobile genetic elements in two of three plasmids, leading to a likely dysfunctional transposon structure. In the third IncF plasmid, a 3,360 bp fragment of Tn*6237* was found, forming a presumable intact and mobile transposon structure flanked by two IS*1R* sequences, which might be mobilizable into other plasmids or the chromosome. The high conjugation frequency of this plasmid has been attributed to the disruption of the *tir* gene, encoding a transfer inhibition protein, by insertion of the Tn*1999* in pOXA-48 (Potron et al., 2014).

Transconjugants showed high plasmid stabilities in the absence of antibiotic pressure. Furthermore, the 63 kb plasmid could also be conjugated to other Enterobacterales species such as *E. cloacae*, *S. marcescens*, or *C. freundii*, indicating a broad host range. This is also evidenced by reports of OXA-48 expressing clinical isolates of different species (Dimou et al., 2012; Chen et al., 2015; Findlay et al., 2017). These properties might lead to persistence of OXA-48 in environmental niches, healthy humans and animals without antibiotic pressure, contributing to further dissemination (Pulss et al., 2018).

Because of the overwhelming epidemiological success of OXA-48, several studies previously suggested that OXA-48 does not only confer resistance to beta-lactams but is also associated with bacterial virulence and fitness (Beyrouthy et al., 2013, 2014; de Toro et al., 2017). To address this hypothesis, the LDH cytotoxicity assay using A549 human lung epithelial cells and the *G. mellonella in vivo* infection model were chosen. These two infection systems have been successfully used to compare virulence across strains from different isolation sites, to identify virulence factors and to analyze the impact of antibiotic resistance genes on fitness and virulence (Insua et al., 2013; Ciesielczuk et al., 2015; Göttig et al., 2016; Tietgen et al., 2018). Even though different virulence properties are analyzed by the LDH assay and the *G. mellonella* infection, a positive correlation between these infection models could be observed for both *E. coli* and *K. pneumoniae* in our study (*R* = 0.43 and *R* = 0.51, respectively).

The clinical isolates were highly diverse in their ability to damage human A549 cells, with LDH release ranging from 16.5 to 96.1% compared to the positive control. *K. pneumoniae* isolates were on average more cytotoxic compared to *E. coli* isolates indicating species-specific virulence (Figure 2). However, EC7215 and KP12536 were the most cytotoxic isolates from the two species. Likewise, these two isolates were highly virulent in the *Galleria* assay. For example, just 8 cfu of EC7215 were able to kill 50% of larvae after 24 h (Figure 3). Beyrouthy et al. (2013, 2014) and de Toro et al. (2017) reported that the presence of virulence genes, e.g., *pap*, *sfa*/*foc*, *hly*, and *afa*/*dra* are correlated with a virulent phenotype when analyzing eight isolates. However, when analyzing 20 isolates neither of these factors correlated with increased virulence or the numbers of virulence genes (Supplementary Figures 4, 5).

Since the dissemination of OXA-48 is not driven by a single clone but rather by the highly prevalent 63 kb-sized IncL plasmid, we investigated the impact of pOXA-48 on virulence and fitness employing isogenic OXA-48-expressing *E. coli* and *K. pneumoniae* transconjugants and parental strains lacking OXA-48. We completely sequenced pOXA-48 but could not identify any genes with known association to virulence or fitness. Yet, 28 ORFs of unknown function were found which hypothetically could have an impact on virulence. However, survival of larvae was not different in transconjugants harboring pOXA-48 compared with the plasmid-free parental strains. Likewise, pOXA-48 carriage did not influence cytotoxicity toward human cells. This is in line with the observation that most of the clinical isolates displayed different virulence phenotypes but harbored the same OXA-48 plasmid. Even though no effect of the 63 kb IncL pOXA-48 was observed in the strains J53 and PRZ, it cannot be excluded that pOXA-48 contributes to virulence in other host strains, since the plasmid-encoded hypothetical ORFs may uniquely interact with other bacterial hosts. Hence, future studies should analyze the impact of pOXA-48 on virulence in additional host strains and also plasmid-cured clinical isolates.

This work has some limitations. Clinical isolates from two large tertiary care hospitals were used, which might not be representative for the whole country. Furthermore, it is not possible to transfer the virulence and fitness data from *in vitro* and *in vivo* model systems directly to a clinical setting. For example, the bacterial isolates may have organ-specific adaptations during infection in patients which cannot be monitored with our infection models, or the uptake of the IncL plasmid into *E. coli* J53 may not accurately recapitulate transmissibility in real clinical settings. The *in vitro* fitness assay allows the comparison of different isolates under standardized conditions. However, the fitness observed is influenced by the choice of media and can therefore not fully comparable to the clinical situation where different nutrient conditions are present and other, more complex factors like the immune system could further influence growth. Nevertheless, to the best of our knowledge this is the most comprehensive study on OXA-48 clinical isolates and plasmids from Germany. Compared to previous studies, we investigated HGT and virulence of OXA-48 producers employing a large number of isolates using *in vitro, in vivo*, and *in silico* methods. Furthermore, we could demonstrate for the first time that the 63 kb IncL plasmid which is disseminated worldwide can be transferred more efficiently compared to IncF plasmids, which might be a reason for its successful dissemination. In addition, acquisition of the 63 kb OXA-48 plasmid had only a minor impact on the fitness of EC-J53 in the competitive fitness assay and also did not lead to impaired growth *in vitro*. This is in contrast to other carbapenemases like NDM-1, which resulted in a highly decreased fitness of EC-J53 (Göttig et al., 2016). The high prevalence and rapid dissemination of OXA-48 can therefore be rather attributed to a low fitness burden and high plasmid stability combined with a highly efficient HGT of the 63 kb IncL plasmid and not increased fitness or virulence.

## Data Availability Statement

The datasets generated for this study can be found at NCBI BioProject accession no. PRJEB27471, PRJEB27468, PRJEB27481, and PRJEB27482.

## Author Contributions

AH, MW, and SG designed the study. JS, CB, YS, FK, FW, TT, SR-C, and JK performed the experiments. JS, MW, CI, AG, and UN carried out sequencing and bioinformatic analyses. AH, JS, and SG analyzed the data and drafted the manuscript. All authors reviewed the manuscript.

## Conflict of Interest

The authors declare that the research was conducted in the absence of any commercial or financial relationships that could be construed as a potential conflict of interest.

## Footnotes

1 https://bigsdb.pasteur.fr/klebsiella/klebsiella.html

2 https://geneious.com

3 http://www.mgc.ac.cn/VFs/

4 https://github.com/tseemann/abricate
