# Supplementary material for: Pathogenicity of Clinical OXA-48 Isolates and Impact of the OXA-48 IncL Plasmid on Virulence and Bacterial Fitness
Source: Front Microbiol. 2019 Nov 1;10:2509. doi: 10.3389/fmicb.2019.02509 (PMC6838017; doi:10.3389/fmicb.2019.02509)
Supplement: Supplementary file 1 [file Data_Sheet_1.docx]

Supplementary Material

Pathogenicity of clinical OXA-48 Isolates and Impact of the OXA‑48 IncL plasmid on Virulence and Bacterial Fitness

Axel Hamprecht, Julian Sommer, Matthias Willmann, Christina Brender, Yvonne Stelzer, Felix F. Krause, Tsvetan Tsvetkov, Florian Wild, Sara Riedel-Christ, Julia Kutschenreuter, Can Imirzalioglu, Aitor Gonzaga, Ulrich Nübel, Stephan Göttig

**Supplementary Table 1.** Primers used in this study.

| **Primer^a^** | **Sequence (5’→ 3’)** | **Target** | **Reference** |
| --- | --- | --- | --- |
| TN_TIR | GTCCATACCCGATGTGATCC | Tn*1999* | This study |
| TN_OXA-48-F | ACAGGGCGTAGTTGTGCTCT |  |  |
| TN_OXA-48-R | AGAGCACAACTACGCCCTGT |  |  |
| TN_pemI | GCCAACCTTCTTCAGTCGAG |  |  |
| L-FW | CGGAACCGACATGTGCCTACT | IncL | (Carattoli et al., 2002) |
| L/M-RV | GAACTCCGGCGAAAGACCTTC |  |  |
| preOXA-48A | TATATTGCATTAAGCAAGGG | *bla*_OXA-48_ | (Potron et al., 2011) |
| preOXA-48B | CACACAAATACGCGCTAACC |  |  |
| Tn6237_2058_F1 | TTCAGCGCAGAAAGACCTGT | Tn*6237* of EC2058 | This study |
| Tn6237_2058_R1 | AGTGCGTTCACCGAATACGT |  |  |
| Tn6237_2058_F2 | TGCAGTTCACTTACACCGCT |  |  |
| Tn6237_2058_R2 | CGATTGATGCGCCGCTTAAA |  |  |
| Tn6237_2196_F1 | ACGTATTCGGTGAACGCACT | Tn*6237* of EC2196 | This study |
| Tn6237_2196_R1 | AGGGCACTGGCAACTAACTC |  |  |
| Tn6237_2196_F2 | TTGTTCTATCCGGACGTGCC |  |  |
| Tn6237_2196_R2 | GGAGCCTGTCATACGCGTAA |  |  |

^a^ Annealing temperature for all primers was 60 °C except for preOXA-48A and preOXA-48B (58 °C).

**Genetic characteristics of OXA-48 *E. coli* (EC) and *K. pneumoniae* (KP) isolates**

All isolates were sequenced using the Illumina MiSeq platform unless otherwise indicated. Sizes of assembled contigs were determined by addition of all contigs >500 bp from final assemblies. Number of contigs refer to all contigs >500 bp of final assemblies. The serotype was determined using the software ABRicate^1^ and the serotype database from SerotypeFinder (Joensen et al., 2015). The *fimH* type was determined by FimTyper (Camacho et al., 2009). Resistance genes were identified using the software ABRicate on the ResFinder database (Zankari et al. 2012). Numbers of virulence genes were calculated using the software ABRicate on the Virulence Factors database (Chen et al., 2016) and additional selected virulence factors, resulting in a collection of 2,769 virulence genes (References 8 – 19). Virulence genes were identified, merged to clusters and classified into functional groups. The following virulence genes were present in all *E. coli* isolates: *foc*A, *yagVWXYZ* (adhesins); *entABCDEFS*, *fepABCDG*, *fes* (Enterobactin); *csgBCDEFG* (fimbria); *cheY*, *flgGH*, *fliGLMP* (flagella); *air*, *celb*, *gcl*, *gnsA*, *malX*, *ompA*, *uidA*, *uspACDEFG*, *wzx*, *ylbE* (miscellaneous); *wzy* (O-antigen); *PAIICFT073* (pathogenicity island); *ykgK*/*ecpR* (regulatory protein); *espL1* (secretion effector); *astA* (toxin).

Capsular type was determined using the software Kaptive (Wick et al., 2018) and the Institute Pasteur database (Brisse et al., 2013). Details are given in Supplementary Tables 2 and 3 and Supplementary Figures 4 and 5.

**Supplementary Table 2** Genetic characteristics of OXA-48 producing *E. coli* (EC) isolates.

| **Isolate** | **Size/bp** | **N50** | **GC %** | **Contigs** | **Serotype** | **fimH Type** | **Beta-lactamase (*bla*) genes**  **encoding** | **Resistance genes** | **Virulence genes (n=)** |
| --- | --- | --- | --- | --- | --- | --- | --- | --- | --- |
| EC1027 | 5,065,938 | 149,098 | 50.69 | 103 | O132:H37 | 38-like | OXA-48, CTX-M-1, TEM-1B | *aadA5*, *dfrA17*, *sul2*, *tet(A)* | 93 |
| EC1639^a^ | 5,271,079 | 5,059,899 | 50.64 | 5 | O15:H1 | 54 | OXA-48, TEM-1B | *aadA5*, *aph(3'')-Ib*, *aph(6)-Id*, *dfrA17*, *mph(A)*, *sul1*, *sul2* | 122 |
| EC2058^a^ | 5,407,964 | 5,407,964 | 50.53 | 1 | O50/O2:H30 | 5 | OXA-48, CTX-M-24, CMY-2, TEM-1B | *aac(3)-IId*, *aadA5*, *dfrA17*, *mph(A)*, *sul1* | 118 |
| EC2196^a^ | 5,289,911 | 5,289,911 | 50.63 | 1 | O50/O2:H30 | 5 | OXA-48, CTX-M-24, TEM-1B | *aac(3)-IId*, *aadA5*, *dfrA17*, *mph(A)*, *sul1* | 119 |
| EC2269^a^ | 5,342,724 | 5,303,872 | 50.62 | 2 | O50/O2:H30 | 5 | OXA-48, CTX-M-24, TEM-1B | *aac(3)-IId*, *aadA5*, *dfrA17*, *mph(A)*, *sul1* | 117 |
| EC2667 | 5,311,655 | 173,999 | 50.47 | 101 | O153:H34 | 58 | OXA-48, CMY-42 | *aph(3'')-Ib*, *aph(6)-Id*, *catA2*, *catB4*, *mph(A)*, *tet(34)*, *tet(A)* | 95 |
| EC2700 | 5,303,963 | 112,841 | 50.48 | 115 | O153:H34 | 58 | OXA-48, CMY-42 | *aph(3'')-Ib*, *aph(6)-Id*, *catA2*, *catB4*, *mph(A)*, *tet(34)*, *tet(A)* | 95 |
| EC3124^a^ | 5,470,285 | 5,405,855 | 50.6 | 4 | O50/O2:H30 | 5 | OXA-48, CTX-M-24, TEM-1B | *aac(3)-IId*, *aadA5*, *dfrA17*, *mph(A)*, *sul1* | 105 |
| EC3239^a^ | 5,636,081 | 5,246,685 | 50.45 | 5 | O16:H48 | 27 | OXA-48 | *tet(34)* | 79 |
| EC3338^a^ | 5,366,215 | 5,121,710 | 50.64 | 6 | O45:H6 | 5 | OXA-48, CTX-M-24, TEM-1B | *mdf(A), tet(34), tet(B)* | 105 |
| EC3428^a^ | 5,234,938 | 2,957,175 | 50.72 | 28 | O50/O2:H30 | 5 | OXA-48, CTX-M-24, TEM-1 | *mph(A)*, *aac(3)-IId*, *sul1*, *aadA5*, *dfrA17* | 116 |
| EC3471 | 4,661,667 | 82,228 | 50.70 | 162 | 09:H4 | 34 | OXA-48, TEM-1B | *qnrS1*, *tet(A)* | 70 |
| EC7215^a^ | 5,036,727 | 4,830,938 | 50.86 | 3 | O160/O8:H10 | 35 | OXA-48, TEM-1B | aadA2, *ant(3'')-Ia*, *aph(3')-Ia*, *aph(3'')-Ib*, *cmlA1*, *dfrA14*, *floR*, *mph(A)*, *sul3*, *tet(A)* | 86 |
| EC9629^a^ | 5,464,324 | 4,397,092 | 50.65 | 49 | O45:H6 | 58 | OXA-48, CTX-M-24, TEM-1B | *tet(34)*, *tet(B)* | 105 |
| EC14604^a^ | 4,979,357 | 4,707,697 | 50.83 | 4 | O9:H4 | - | OXA-48, CTX-M-15, TEM-1B | *dfrA14*, *erm(B)*, *mph(A)*, *tet(34)*, *tet(B)* | 86 |

^a^ Isolate additionally sequenced by SMRT.

**Supplementary Table 3** Genetic characteristics of OXA-48 producing *K. pneumoniae* (KP) isolates.

| **Isolate** | **Size/bp** | **N50** | **GC %** | **Contigs** | **Capsular type (wzi)** | **Capsular type (Kaptive)** | **Beta-lactamase (*bla*) genes**  **encoding** | **Resistance genes** | **Virulence genes (n=)** |
| --- | --- | --- | --- | --- | --- | --- | --- | --- | --- |
| KP659 | 5,582,193 | 327,781 | 57.34 | 109 | 93 | KL112:O1v1 | OXA-48, CTX-M-15, SHV-28, TEM‑1B, OXA-1 | *aac(3)-IIa*, *aac(6')Ib-cr*, *aph(3'')-Ib*, *aph(6)-Id*, *catB4*, *fosA6*, *oqxA*, *oqxB*, *sul2*, *tet(A)* | 44 |
| KP920^a^ | 5,590,823 | 5,231,183 | 57.30 | 4 | 50 | KL15:O3b | OXA-48, CTX-M-15, SHV-1, TEM-1B, OXA-1 | *aac(6')Ib-cr*, *aadA2*, *ant(3'')-Ia* *catB4*, *dfrA12*, *fosA5*, *mph(A)*, *sul1*, *tet(A)* | 42 |
| KP980^a^ | 5,553,749 | 5,216,735 | 57.32 | 4 | 50 | KL15:O3b | OXA-48, CTX-M-15, SHV-1, TEM-1B, OXA-1 | *aac(6')Ib-cr*, *aadA2*, *ant(3'')-Ia* *catB4*, *dfrA12*, *fosA5*, *mph(A)*, *sul1*, *tet(A)* | 42 |
| KP1151 | 5,616,301 | 241,362 | 57.41 | 109 | 24 | KL24:O1 | OXA-48, CTX-M-15, SHV-106, OXA-1 | *aac(3)-IIa*, *aac(6')Ib*, *catB4*, *dfrA14*, *fosA6*, *oqxA*, *oqxB*, *tet(D)*, *qnrS1* | 44 |
| KP1664 | 5,497,404 | 284,915 | 57.35 | 70 | 50 | KL15:O3b | OXA-48, CTX-M-15, SHV-1, TEM-1B, OXA-1 | *aac(6')Ib-cr*, *aadA2*, *catB4*, *dfrA12*, *fosA5*, *mph(A)*, *oqxA*, *oqxB*, *sul1*, *tet(A)* | 42 |
| KP1673 | 5,558,274 | 240,905 | 57.00 | 91 | 137 | KL17:O1v1 | OXA-48, CTX-M-15, SHV-1, TEM-1A, OXA-1, OXA‑9 | *aac(3)-IIa*, *aac(6')Ib-cr*, *catB4*, *dfrA14*, *fosA*, *tet(D)* | 54 |
| KP1696 | 5,672,407 | 280,648 | 57.02 | 61 | 173 | KL102:O2v2 | OXA-48, CTX-M-15, SHV-110, TEM‑1B, OXA-1 | *aac(3)-IIa*, *aac(6')Ib*, *ant(3'')-Ia*, *catB*, *dfrA15*, *fosA*, *oqxA*, *oqxB*, *sul1* | 55 |
| KP1766 | 5,481,479 | 170,472 | 57.07 | 115 | 137 | KL17:O1v1 | OXA-48, CTX-M-15, SHV-1, TEM-1A, OXA-1 | *aac(3)-IIa*, *aac(6')Ib-cr* *catB4* *dfrA14*, *fosA*, *oqxA*, *oqxB*, *tet(D)* | 54 |
| KP1982 | 5,641,317 | 126,169 | 57.02 | 106 | 173 | KL102:O2v2 | OXA-48, CTX-M-15, SHV-110, TEM‑1B, OXA-1 | *aac(3)-IIa*, *aac(6')Ib-cr*, *catB4*, *dfrA15*, *fosA*, *oqxA*, *oqxB*, *sul1* | 55 |
| KP2255 | 5,285,767 | 274,273 | 57.49 | 57 | 109 | KL36:O4 | OXA-48, CTX-M-15, SHV-11, OXA-1 | *aac(6')Ib-cr*, *aadA2*, *ant(3'')-Ia* *armA*, *catB4*, *dfrA12*, *fosA6*, *mph(E)*, *msr(E)*, *sul1* | 42 |
| KP2451 | 5,563,511 | 167,004 | 57.09 | 79 | 187 | KL27:O4 | OXA-48, CTX-M-15, SHV-11, TEM-1B | *aac(3)-IIa*, *aac(6')Ib-cr*, *aph(3'')-Ib*, *aph(6)-Id*, *catB4*, *dfrA14*, *fosA*, *oqxA*, *oqxB*, *sul2, tet(A)*, *qnrB1* | 43 |
| KP2540 | 5,504,911 | 327,781 | 57.34 | 82 | 50 | KL15:O3b | OXA-48, SHV-1, TEM-1B, OXA-1 | *aac(6')Ib-cr*, *aadA2*, *catB4*, *dfrA12*, *fosA5*, *mph(A)*, *oqxA*, *oqxB*, *sul1*, *tet(A)* | 42 |
| KP2575 | 5,655,984 | 381,474 | 57.32 | 56 | 259 | KL20:O3a | OXA-48, LEN24 | *fosA* | 42 |
| KP2670 | 5,570,556 | 195,888 | 57.16 | 70 | 137 | KL17:O1v1 | OXA-48, SHV-1 | *fosA*, *oqxA*, *oqxB* | 55 |
| **Isolate** | **Size/bp** | **N50** | **GC %** | **Contigs** | **Capsular type (wzi)** | **Capsular type (Kaptive)** | **Beta-lactamase (*bla*) genes**  **encoding** | **Resistance genes** | **Virulence genes (n=)** |
| KP2746 | 5,573,327 | 80,814 | 57.18 | 142 | 137 | KL17:O1v1 | OXA-48, SHV-1 | *catB4*, *fosA* | 55 |
| KP3379 | 5,305,651 | 241,362 | 57.41 | 68 | 173 | KL102:O2v2 | OXA-48, SHV-28 | *fosA6*, *oqxA*, *oqxB* | 42 |
| KP3907 | 5,464,222 | 200,612 | 57.25 | 79 | 173 | KL102:O2v2 | OXA-48, CTX-M-15, SHV-28, TEM‑1B, OXA-1 | *aac(3)-IIa*, *aac(6')Ib-cr*, *aph(3'')-Ib*, *aph(6)-Id*, *catB4*, *dfrA14*, *fosA6*, *oqxA*, *oqxB*, *sul2*, *tet(A)*, *qnrB1* | 42 |
| KP12536^a^ | 5,387,277 | 5,323,688 | 57.40 | 2 | 298 | KL51:O12 | OXA-48, SHV-1 | *fosA* | 43 |
| KP12883 | 5,427,158 | 371,809 | 57.35 | 43 | 262 | KL21:O3b | OXA-48, SHV-99 | *fosA6*, *fosA7* | 44 |
| KP13815 | 5,638,988 | 194,818 | 57.14 | 103 | 24 | KL24:O2v1 | OXA-48, CTX-M-15, SHV-1, OXA-1 | *aac(3)-IIa*, *aac(6')Ib-cr*, *aadA5*, *catA1*, *catB4*, *dfrA17*, *mph(A)*, *oqxA*, *oqxB*, *sul1* | 59 |

^a^ Isolate additionally sequenced by SMRT.

**
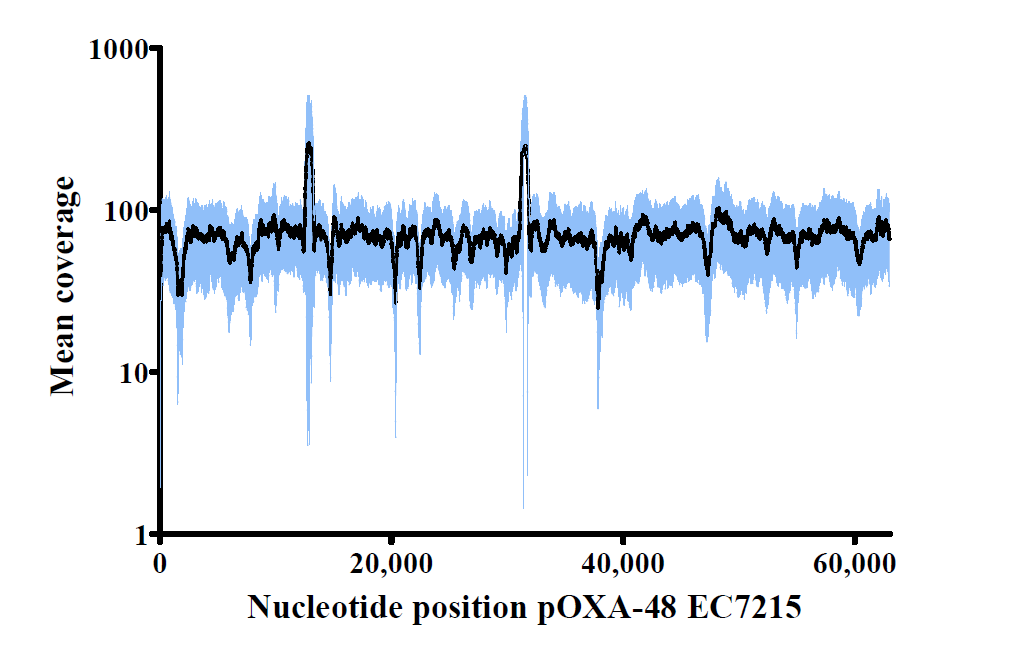
**

**Supplementary Figure 1** Mean coverage (black line) and standard deviation (blue) of short reads of 21 isolates harboring the 63 kb *bla*_OXA-48_ encoding IncL plasmid compared to the complete nucleotide sequence of the 63 kb IncL plasmid from strain EC7215 (Accession No. LR025098.1). The coverage peaks at position 13,000 and 32,000 indicate insertion elements in the reference sequence.

**
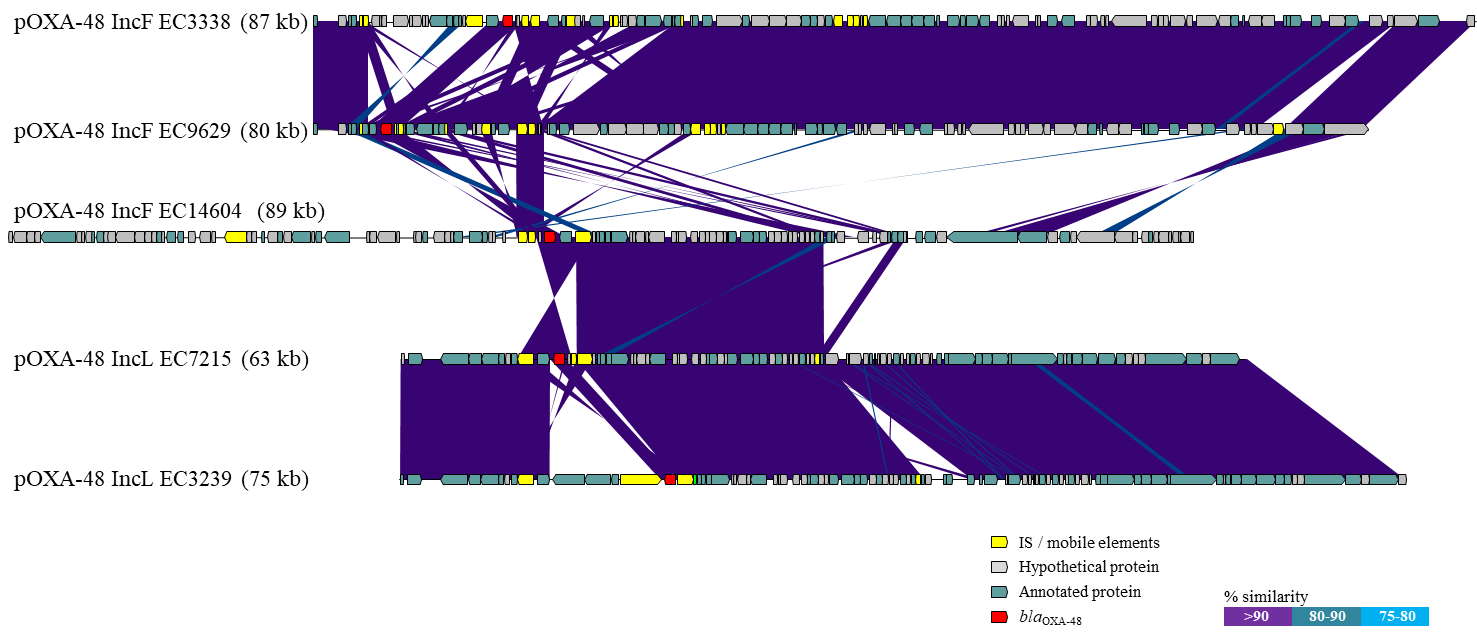
**

**Supplementary Figure 2** Comparison of five plasmid nucleotide sequences using BLAST and a custom Perl script for plotting the alignment. Colored arrows indicate *bla*_OXA-48_ (red), Insertion sequences (IS) or mobile elements (yellow), genes encoding for proteins of unknown function (gray) and genes encoding for annotated proteins (light blue). The plasmid sequences from EC14604, EC3338 and EC9629 showed highest coverage and sequence identities to CP032226.1, CP018954.1 and CP018954.1, respectively.

**Supplementary Figure 3** Transconjugation frequencies of pOXA-48 employing different Enterobacterales acceptor species. The *K. pneumoniae* clinical isolate KP980 carrying the standard 63 kb IncL *bla*_OXA-48_ plasmid was used as a donor.


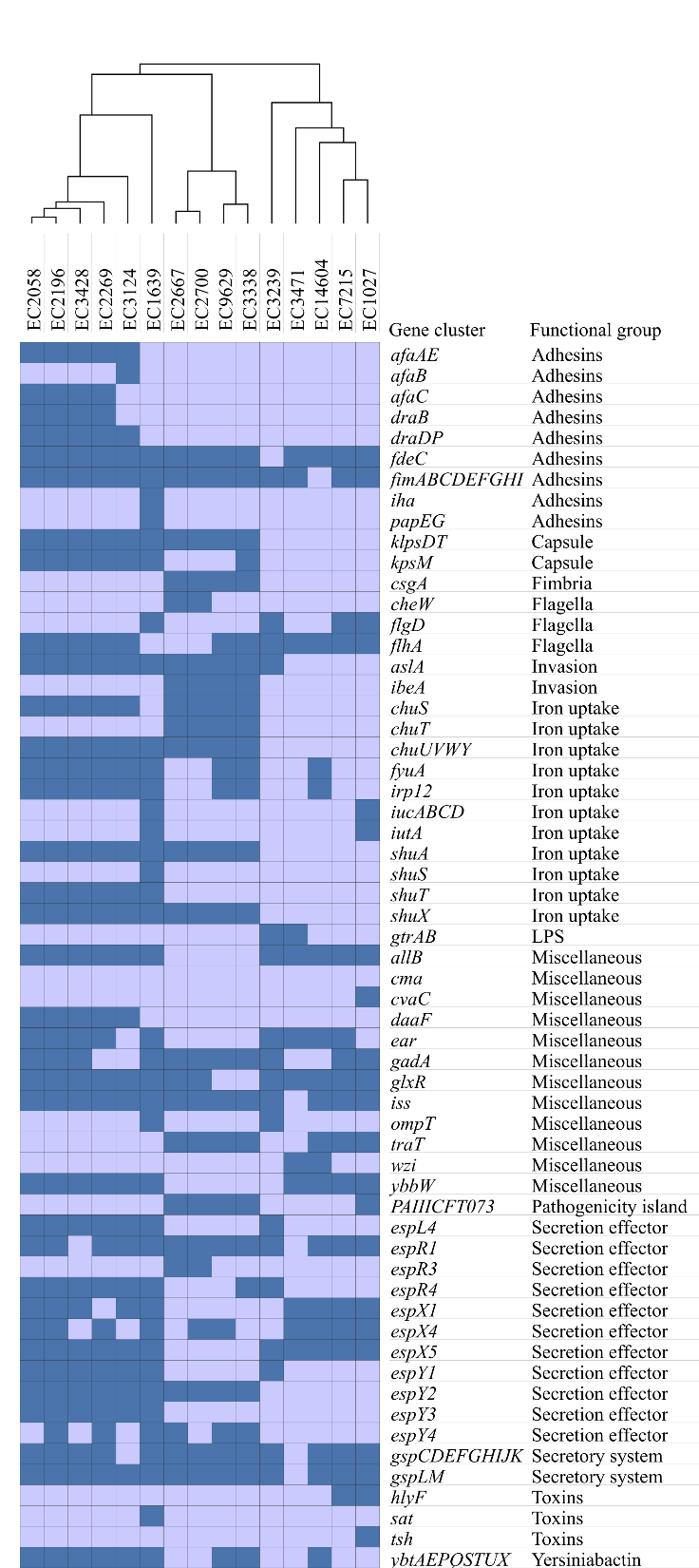


**Supplementary Figure 4** Presence of virulence genes in WGS data of *E. coli* with isolates ordered by hierarchical clustering*.*


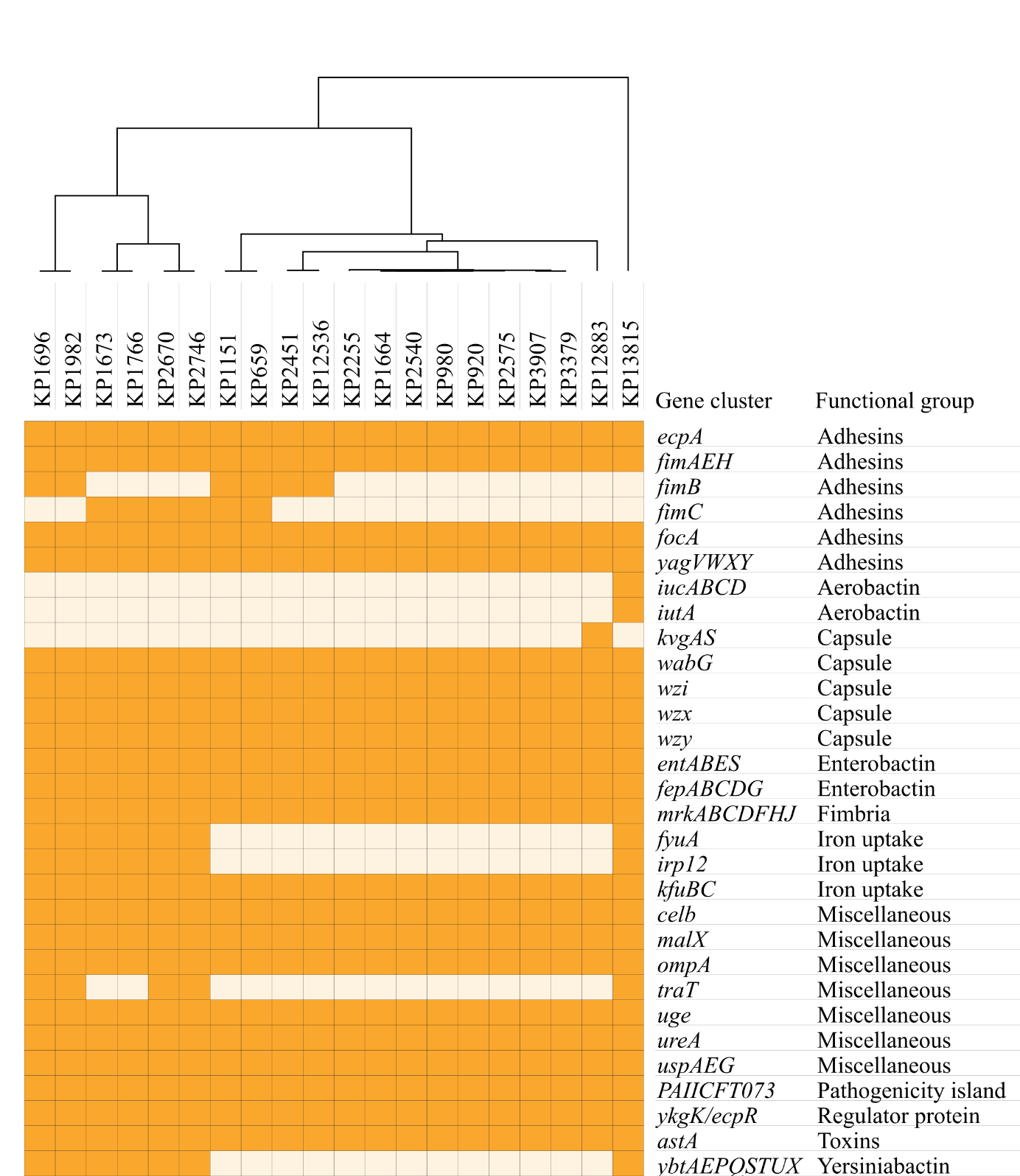


**Supplementary Figure 5** Presence of virulence genes in WGS data of *K. pneumoniae* with isolates ordered by hierarchical clustering*.*

**References**

1. Carattoli, A., Seiffert, S. N., Schwendener, S., Perreten, V., and Endimiani, A. (2002). Differentiation of IncL and IncM Plasmids Associated with the Spread of Clinically Relevant Antimicrobial Resistance. *PLoS One* 10:e0123063. doi: 10.1371/journal.pone.0123063
2. Potron, A., Nordmann, P., Lafeuille, E., Al Maskari, Z., Al Rashdi, F., and Poirel, L. (2011).Characterization of OXA-181, a carbapenem-hydrolyzing class D beta-lactamase from Klebsiella pneumoniae. *Antimicrob Agents Chemother* 55, 4896-4899.
3. Joensen, K. G., Tetzschner, A. M., Iguchi, A., Aarestrup, F. M., and Scheutz, F. (2015). Rapid and easy in silico serotyping of Escherichia coli using whole genome sequencing (WGS) data. *J.Clin.Microbiol* 53, 2410-2426.
4. Camacho, C., Coulouris, G., Avagyan, V., Ma, N., Papadopoulos, J., Bealer, K., and Madden, T. L. (2009) BLAST+: architecture and applications. *BMC Bioinformatics* 10, 421.
5. Zankari, E., Hasman, H., Cosentino, S., Vestergaard, M., Rasmussen, S., Lund, O., Aarestrup, F. M., and Larsen, M. V., (2012). Identification of acquired antimicrobial resistance genes. *J Antimicrob Chemother* 67,2640-2644.
6. Chen, L. H., Zheng, D. D., Liu, B., Yang, J., and Jin, Q. (2016). VFDB 2016: hierarchical and refined dataset for big data analysis-10 years on. [*Nucleic Acids Res* 44, D694-D697](http://www.ncbi.nlm.nih.gov/pubmed/26578559). doi: 10.1093/nar/gkv1239
7. Tietgen, M., Semmler, T., Riedel-Christ, S., Kempf, V.A.J., Molinaro, A., Ewers, C., and Göttig, S. (2018). Impact of the colistin resistance gene mcr-1 on bacterial fitness. *Int J Antimicrob Agents* 51, 554-561.
8. Brisse, S., Fevre, C., Passet, V., Issenhuth-Jeanjean, S., Tournebize, R., Diancourt, L., and Grimont P. (2013). Virulent Clones of Klebsiella pneumoniae: Identification and Evolutionary Scenario Based on Genomic and Phenotypic Characterization. *PLoS One* 4:e4982. doi: 10.1371/journal.pone.0004982
9. Beyrouthy, R., Robin, F., Cougnoux, A., Dalmasso, G., Darfeuille-Michaud, A., Mallat, H., Dabboussi, F., Hamzé, M., and Bonnet, R. (2013). Chromosome-mediated OXA-48 carbapenemase in highly virulent Escherichia coli. *Antimicrob Chemother* 68, 1558-1567.
10. Beyrouthy, R., Robin, F., Dabboussi, F., Mallat, H., Hamzé, M., and Bonnet, R. (2014). Carbapenemase and virulence factors of Enterobacteriaceae in North Lebanon between 2008 and 2012: evolution via endemic spread of OXA-48. *J Antimicrob Chemother* 69, 2699-2705.
11. Williamson, D. A., Mills, G., Johnson, J. R., Porter, S., and Wiles, S. (2014). In vivo correlates of molecularly inferred virulence among extraintestinal pathogenic Escherichia coli (ExPEC) in the wax moth Galleria mellonella model system. *Virulence* 5, 388–393.
12. Wu, K. M., Li, L. H., Yan, J. J., Tsao, N., Liao, T. L., Tsai, H. C., Fung, C. P., Chen, H. J., Liu, Y. M., Wang, J. T., Fang, C. T., Chang, S. C., Shu, H. Y., Liu, T. T., Chen, Y. T., Shiau, Y. R., Lauderdale, T. L., Su, I. J., Kirby, R., and Tsai, S. F. (2009). Genome sequencing and comparative analysis of Klebsiella pneumoniae NTUH-K2044, a strain causing liver abscess and meningitis. *Journal of bacteriology* 191, 4492-4501
13. Johnson, J. R., Porter, S. B., Zhanel, G., Kuskowski, M. A., and Denamurd, E. (2012). Virulence of Escherichia coli Clinical Isolates in a Murine Sepsis Model in Relation to Sequence Type ST131 Status, Fluoroquinolone Resistance, and Virulence Genotype. *Infect Immun* 80, 1554–1562.
14. Lee, I. R., Molton, J. S., Wyres, K. L., Gorrie, C., Wong, J., Hoh, C. H., Teo, J., Kalimuddin, S., Lye, D. C., Archuleta, S., Holt, K. E. and Gan, Y. H. (2016). Differential host susceptibility and bacterial virulence factors driving Klebsiella liver abscess in an ethnically diverse population. *Sci Rep* 6, 29316. doi: 10.1038/srep29316
15. Srinivasan, U., Foxman, B., and Marrs, C. F. (2003). Identification of a Gene Encoding Heat-Resistant Agglutinin in Escherichia coli as a Putative Virulence Factor in Urinary Tract Infection. *J Clin Microbiol 41* ,285-289.
16. Lafeuille, E., Decré, D., Mahjoub-Messai, F., Bidet, P., Arlet, G., and Bingen, E. (2013). OXA-48 carbapenemase-producing Klebsiella pneumoniae isolated from Libyan patients. *Microb Drug Resist* 19, 491-497.
17. Frömmel, U., Lehmann, W., Rödiger, S., Böhm, A., Nitschke, J., Weinreich, J., Groß, J., Roggenbuck, D., Zinke, O., Ansorge, H., Vogel, S., Klemm, P., Wex, T., Schröder, C., Wieler, L. H., and Schierack, P. (2013). Adhesion of human and animal Escherichia coli strains in association with their virulence-associated genes and phylogenetic origins. *Appl Environ Microbiol* 79, 5814-5829.
18. Brisse, S., Fevre, C., Passet, V., Issenhuth-Jeanjean, S., Tournebize, R., Diancourt, L., Grimont, P. (2009). Virulent clones of Klebsiella pneumoniae: identification and evolutionary scenario based on genomic and phenotypic characterization. *PLoS One* 4:e4982. doi: 10.1371/journal.pone.0004982
19. Liu, Y., Li, X. Y., Wan, L. G., Jiang, W. Y., Yang, J. H., and Li, F. Q. (2014). Virulence and transferability of resistance determinants in a novel Klebsiella pneumoniae sequence type 1137 in China. *Microb Drug Resist* 20, 150-155.
20. Wick, R. R., Heinz, E., Holt, K. E., and Wyres, K. L. (2018). Kaptive Web: User-Friendly Capsule and Lipopolysaccharide Serotype Prediction for Klebsiella Genomes*. J.Clin.Microbiol* 25:e00197-18.
21. Brisse, S., Passet, V., Haugaard, A. B., Babosan, A., Kassis-Chikhani, N., Struve, C., and Decré, D. (2013). wzi Gene sequencing, a rapid method for determination of capsular type for Klebsiella strains. *J Clin Microbiol*. 51, 4073-4078.
